# Screening potential immune signatures for early-stage basal-like/triple-negative breast cancer

**DOI:** 10.1186/s12957-022-02683-2

**Published:** 2022-06-24

**Authors:** Min Wu, Keyu Yuan, Shuzhen Lyu, Yanping Li

**Affiliations:** Galactophore Department, Galactophore Center, Beijing Shijitan Hospital, Capital Medical University, Tieyi Road 10, Haidian District, Beijing, 100038 China

**Keywords:** Breast cancer, Triple negative, Basal-like, Early stage, Immune signature

## Abstract

**Background:**

Breast cancer (BC) is a highly heterogeneous disease. Among the BC molecular subtypes, basal-like/triple-negative BC (TNBC) is characterized by a high propensity for relatively early metastases and a lack of available endocrine and targeted therapies. Therefore, this study aimed to discover potential signatures for predicting the immune response in early-stage basal-like/triple-negative BC.

**Method:**

A total of 86 cases of early-stage TNBC from the TCGA and 459 cases of normal breast tissue from GTEx were enrolled and analyzed to screen out differentially expressed genes (DEGs). Then, the prognostic effect and tumor immune cell infiltration relationship with the basal-like-specific DEGs were also evaluated.

**Results:**

A total of 1556 DEGs, including 929 upregulated genes and 627 downregulated genes, were screened in early-stage basal-like BC. Two prognosis-associated DEGs, GAL and TTC36, were finally found to be basal-like BC specific. However, only GAL was significantly correlated with tumor immune-infiltrating cells, especially CD8^+^ T cells. The expressions of GAL and TTC36 were revalidated by using the GEO dataset.

**Conclusion:**

GAL might be an immune signature for the response to immune checkpoint therapy in early basal-like/triple-negative BC.

**Supplementary Information:**

The online version contains supplementary material available at 10.1186/s12957-022-02683-2.

## Introduction

Breast cancer is a major cancer type that occurs in women around the world, especially in transitioned countries [1]. In females, breast cancer (BC) accounts for 31% of new cancers in all sites and accounts for 15% of cancer deaths in the USA [2]. In detail, cancer statistics predicted that 287,850 new female BC patients would be diagnosed in 2022 in the USA and 43,250 of them would die [2]. The hormonal and reproductive risk factors for female BC mainly include lack of breastfeeding, later age at menopause, and early age at menarche [3]. The other risk factors are obesity, alcohol intake, and BRCA mutations [4].

BC mortality shows a slow decline, but the incidence has steadily increased in recent years, especially in transitioning countries, such as Asia, South America, and Africa [5, 6]. The 5-year relative survival rate of BC in females has recently been approximately 85–90% in high-income countries, which is one of the highest rates among all cancers [7]. This is mainly attributed to mammography screening and improving therapies. BC is a highly heterogeneous disease, and the different pathological subtypes of BC have obviously different treatment responses and long-term outcomes.

Based on the expression of hormonal receptors (HR) (including estrogen and progesterone receptors, ER and PR) and human epidermal growth factor receptor 2 (HER2), BC can be classified into four subgroups: luminal A, luminal B, HER2 overexpression, and triple-negative breast cancer [8]. According to microarray-based gene expression profiling, BC can be divided into five intrinsic subtypes: luminal A, luminal B, HER2-positive enriched, basal-like, and normal-like [9]. Among these subtypes, the basal-like type accounts for approximately 10–20% of all BCs [10]. Basal-like BC, which originates from myoepithelial cells of the breast duct epithelium, has no expression of ER, PR, or HER2. The basal-like BC overlapped in approximately 80% of TNBCs [11]. Basal-like BC has the characteristics of high cell proliferation, high propensity for earlier metastases, poor histopathological grade, and a lack of endocrine and targeted therapies.

The standard therapies for basal-like BC are surgical resection and systemic chemotherapy. Thus, the long-term prognosis for basal-like BC remains poor. Exploring the molecular targets or biomarkers that help improve therapeutic efficacy is still a challenge. Due to the characteristics of a high tumor mutation load and abundant immune cell infiltration in basal-like/triple-negative BC, immunotherapy for triple-negative BC has been extensively investigated and recommended by the guidelines of the national comprehensive cancer network [12, 13]. Immunotherapy drugs for TNBC mainly include programmed cell death protein 1 (PD-1)/PD-L1 inhibitors, such as pembrolizumab and atezolizumab [14]. However, the PD-1/PD-L1 inhibitors have a low response rate (approximately 5%) in the treatment of basal-like/triple-negative BC. The response rate could be increased to 20% in PD-L1-positive basal-like/triple-negative BC [15]. Thus, there is still a lack of predictive markers for the efficacy of immunotherapy in basal-like/triple-negative BC.

Therefore, this study aimed to discover potential signatures for predicting the immune response in early-stage basal-like BC.

## Methods

### Identifying differentially expressed genes (DEGs) in early-stage basal-like BC

The RNA-sequencing expression profiles and clinical data of BC were obtained from the TCGA. The data of early-stage basal-like BC were screened and analyzed in a further step. In this study, the early stage of BC was defined as female patients diagnosed at American Joint Committee on Cancer TNM Staging System (AJCC TNM, 2018 Edition) stages I and IIA. The gene expression data of normal breast tissues were obtained from GTEx as the control. The DEGs were screened by comparing the gene expression of early-stage basal-like BC and normal breast tissue on R software version v4.0.3 (The R Foundation for Statistical Computing, 2020) using the LIMMA package. Genes with an adjusted *P* value < 0.05 and |Log2 FC| ≧ 2 were considered as DEGs. The functional analysis of DEGs was also performed using DAVID Bioinformatics Resources (2021 Update) (https://david.ncifcrf.gov/). The protein–protein interaction (PPI) network of DEGs was constructed by applying the STRING dataset (https://cn.string-db.org/) which was performed on Cytoscape version 3.7.2 software [16]. The minimum required interaction score was set as 0.70 for high confidence.

### Overall survival (OS) analysis and BC molecular subtype-specific DEGs

The prognostic effect of DEGs in early-stage basal-like BC was analyzed using R software version v4.0.3 (The R Foundation for Statistical Computing, 2020) using the log-rank test. The DEGs with a *P* value < 0.05 were considered to be prognostically significant. To screen the basal-like BC-specific DEGs, the obtained prognostic DEGs were explored in GEPIA2 (http://gepia2.cancer-pku.cn/#analysis).

### Expression validation of basal-like BC-specific DEGs

To verify the expression of the identified basal-like-specific DEGs, the gene expression profiles of GSE135565 and GSE42568 from GEO datasets (https://www.ncbi.nlm.nih.gov/geo) were analyzed. GSE135565 [17] contained 84 cases of TNBC, which included 29 cases of early-stage TNBC. The gene profiles of 17 normal breast tissues included in GSE42568 [18] were used as controls in this study. An unpaired *t* test was performed using GraphPad Prism version 5.01 (GraphPad Software, Inc.) to compare the differences between groups, and a *P* value < 0.05 was considered significant.

### Immune infiltration analysis of prognostic DEGs

To explore the immune function of the prognostic DEGs in early-stage basal-like BC, immune infiltration analysis was performed using R software version v4.0.3 (The R Foundation for Statistical Computing, 2020), and the pheatmap package was used to draw the correlations between gene expression and immune score. Spearman’s correlation analysis was applied, and a *P* value < 0.05 was considered significant. The expression distribution of eight immune checkpoint-related genes, namely, *SIGLEC15*, *TIGIT*, *CD274*, *HAVCR2*, *PDCD1*, *CTLA4*, *LAG3*, and *PDCD1LG2*, was determined in early-stage basal-like BC. The Wilcoxon test was applied to compare the difference between the high and low expression of basal-like BC-specific DEG groups. A *P* value < 0.05 was considered significant. The correlation between prognostic DEGs and CD8^+^ T cells in the BC molecular subtypes was analyzed on the TIMER2.0 website (http://timer.cistrome.org/).

## Results

### Screening DEGs in early-stage basal-like BCs

**Fig. 1:**
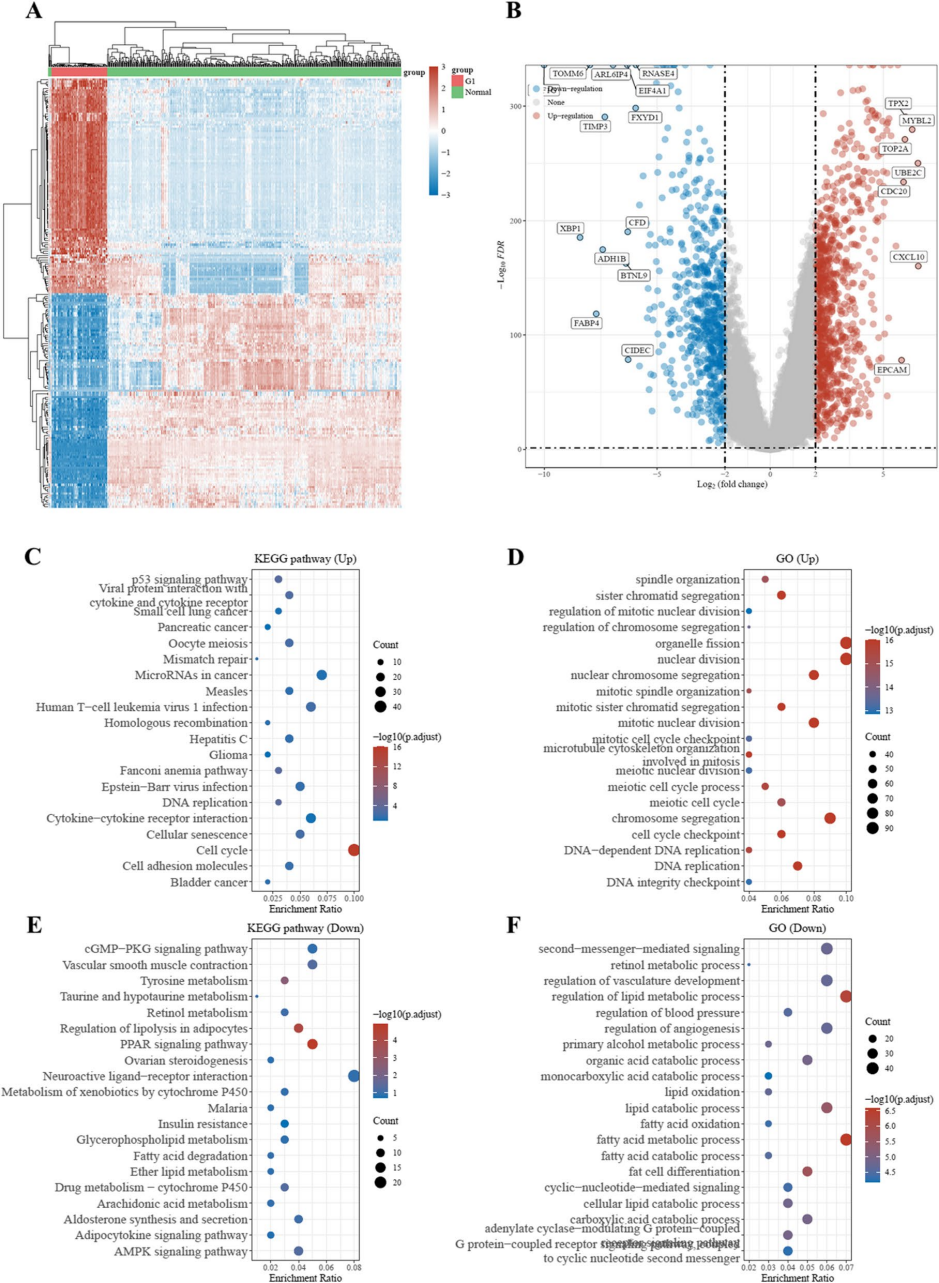
Screening and functional analysis of differentially expressed genes in early-stage basal-like/triple-negative breast cancer. **A** Heatmap of gene expression. **B** Volcano plot of differentially expressed genes. **C**,** D** The KEGG and GO results of the upregulated differentially expressed genes. **E**,** F** The KEGG and GO results of the downregulated differentially expressed genes. G1, early-stage basal-like BC; GO, gene oncology

**Fig. 2:**
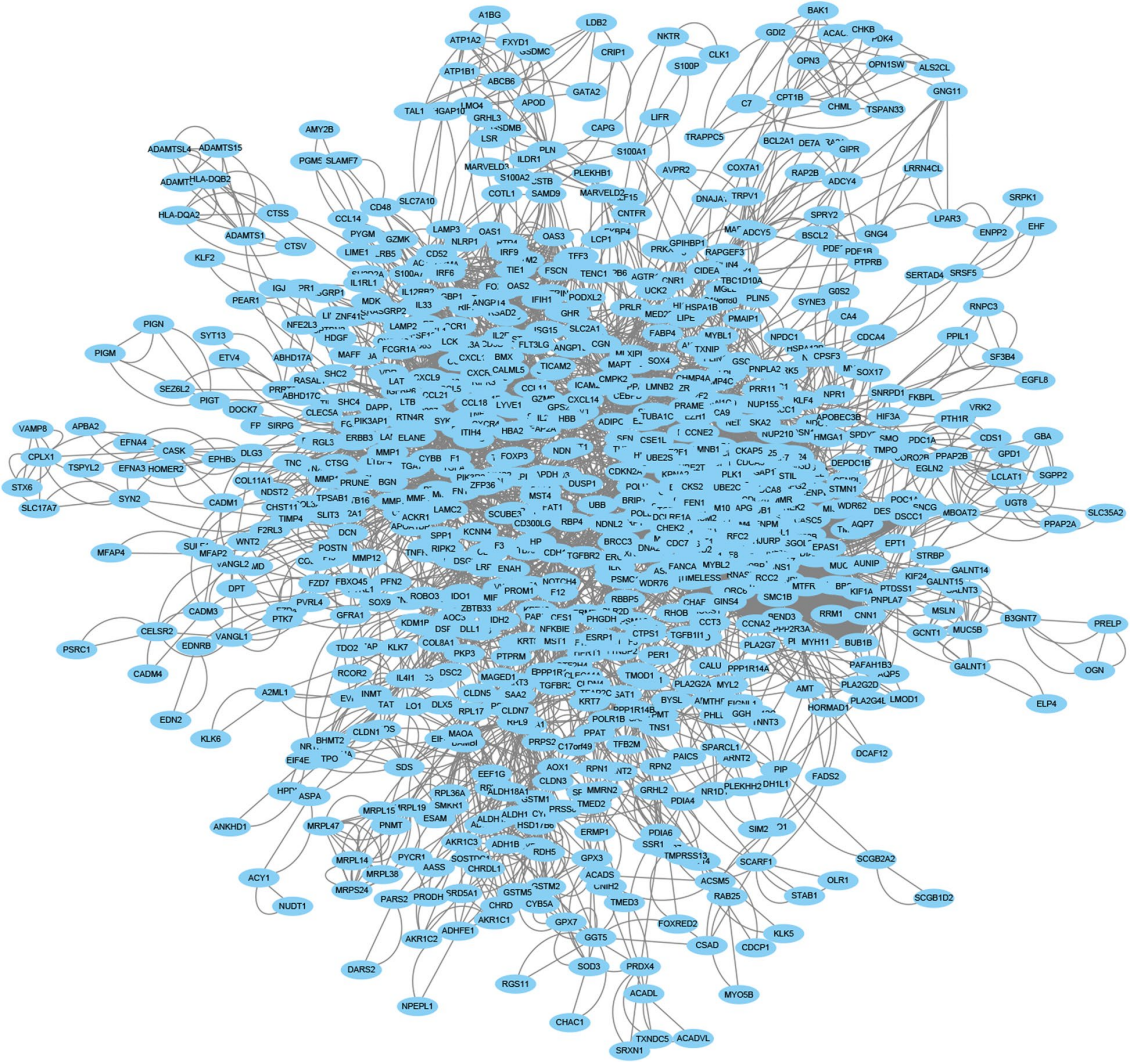
The protein–protein interaction network of the differentially expressed genes in early-stage basal-like/triple-negative breast cancer

The RNA-sequencing expression profiles of 86 cases of early-stage basal-like BC and 459 cases of normal breast tissues were included and explored in this study. A total of 1556 DEGs were identified, with 929 genes upregulated and 627 genes downregulated (Fig. 1A). The heatmap and volcano plot are shown in Fig. 1B. The results of functional analysis revealed that the upregulated DEGs were mainly involved in nuclear division, organelle fission, chromosome segregation, and cell cycle checkpoint, while the downregulated DEGs were mainly involved in the regulation of lipid metabolic processes, second messenger-mediated signaling, and fatty acid metabolic processes (Fig. 1C). The results of the KEGG pathway analysis showed that the upregulated DEGs were mostly involved in the cell cycle, the p53 signaling pathway, and DNA replication, with the downregulated DEGs mostly involved the PPAR signaling pathway, the regulation of lipolysis in adipocytes, and tyrosine metabolism (Fig. 1D). The PPI network of the above DEGs was also built as shown in Fig. 2. The average node degree was 10.5. The avg. local clustering coefficient was 0.4, and the PPI enrichment *P* value was less than 1.0e − 16.

### Overall survival analysis and identification of basal-like BC-specific DEGs

**Fig. 3:**
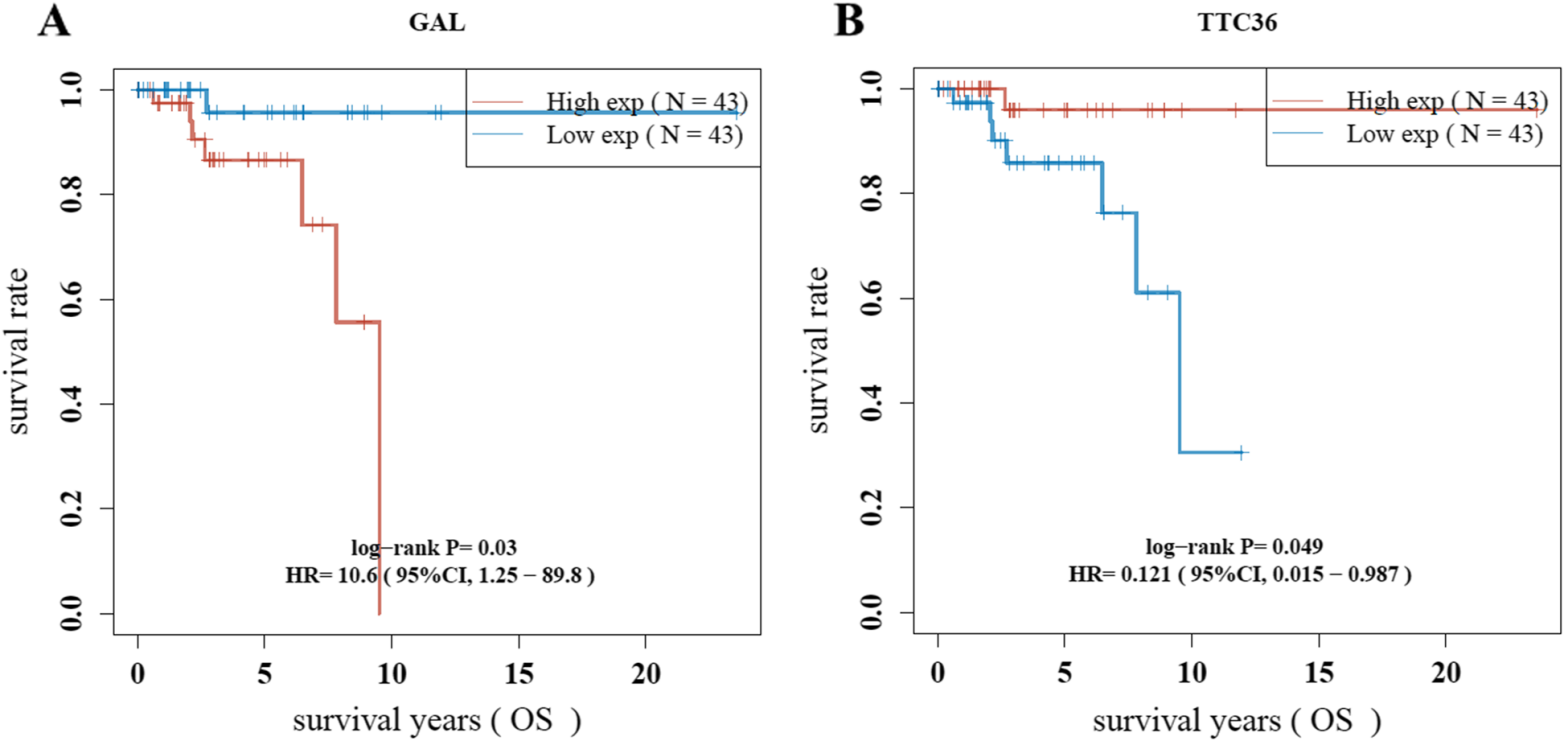
Overall survival analysis of GAL and TTC36 in early-stage basal-like/triple-negative breast cancer. **A** GAL expression. **B** TTC36 expression. N, number; HR, hazard ratio; CI, confidence interval

The OS analysis results showed that the only 22 DEGs were significantly related to the prognosis in early-stage basal-like BCs, namely, trophinin-associated protein (TROAP); claudin 7 (CLDN7); growth arrest specific 2-like 3 (GAS2L3); galanin and GMAP prepropeptide (GAL); Zyg-11 family member A, cell cycle regulator (ZYG11A); uridine-cytidine kinase 2 (UCK2); anillin actin binding protein (ANLN); phospholipid phosphatase 1 (PLPP1); PGAM family member 5, mitochondrial serine/threonine protein phosphatase (PGAM5); grainyhead-like transcription factor 2 (GRHL2); family with sequence similarity 83 member D (FAM83D); CHYLS1 centriolar and ciliogenesis associated (HYLS1); solute carrier family 7 member 5 (SLC7A5); Interleukin 33 (IL33); centromere protein E (CENPE); tyrosine 3-monooxygenase/tryptophan 5-monooxygenase activation protein zeta (YWHAZ); cytochrome c oxidase assembly factor 7 (COA7); LIF receptor subunit alpha (LIFR); extra spindle pole bodies like 1, separase (ESPL1); PARP1 binding protein (PARPBP); tetratricopeptide repeat domain 36 (TTC36); and Dedicator of cytokinesis 6 (DOCK6) (Table 1, Fig. 3).

**Table 1:** Overall survival analysis results of differentially expressed genes in early-stage basal-like/triple-negative breast cancer

Genes	*P* value	HR	Low 95% CI	High 95% CI
TROAP	0.025391	11.58053	1.352293	99.17133
CLDN7	0.028033	10.57329	1.289517	86.69483
GAS2L3	0.030351	10.3481	1.248294	85.78358
GAL	0.030437	10.5947	1.249694	89.82009
ZYG11A	0.03095	10.42861	1.239709	87.72707
UCK2	0.033021	10.17053	1.205618	85.79805
ANLN	0.033041	9.89309	1.20264	81.38202
PLPP1	0.034681	0.10048	0.011913	0.847494
PGAM5	0.036512	9.426576	1.151162	77.19189
GRHL2	0.038303	9.417034	1.128453	78.58594
FAM83D	0.038744	9.148677	1.121338	74.64144
HYLS1	0.039237	9.069905	1.114938	73.78271
SLC7A5	0.039748	9.121387	1.109184	75.00985
IL33	0.041652	0.112615	0.013774	0.920752
CENPE	0.042031	8.895662	1.081924	73.14081
YWHAZ	0.043559	8.711763	1.064576	71.29114
COA7	0.044402	8.712899	1.055671	71.91126
LIFR	0.04678	0.118868	0.014565	0.970122
ESPL1	0.047336	8.37144	1.025295	68.35204
PARPBP	0.048269	8.307202	1.016239	67.90686
TTC36	0.048538	0.120893	0.014815	0.986523
DOCK6	0.049538	8.280691	1.004277	68.27783

*HR* hazard ratio, *CI* confidence interval, *BC* breast cancer

**Fig. 4:**
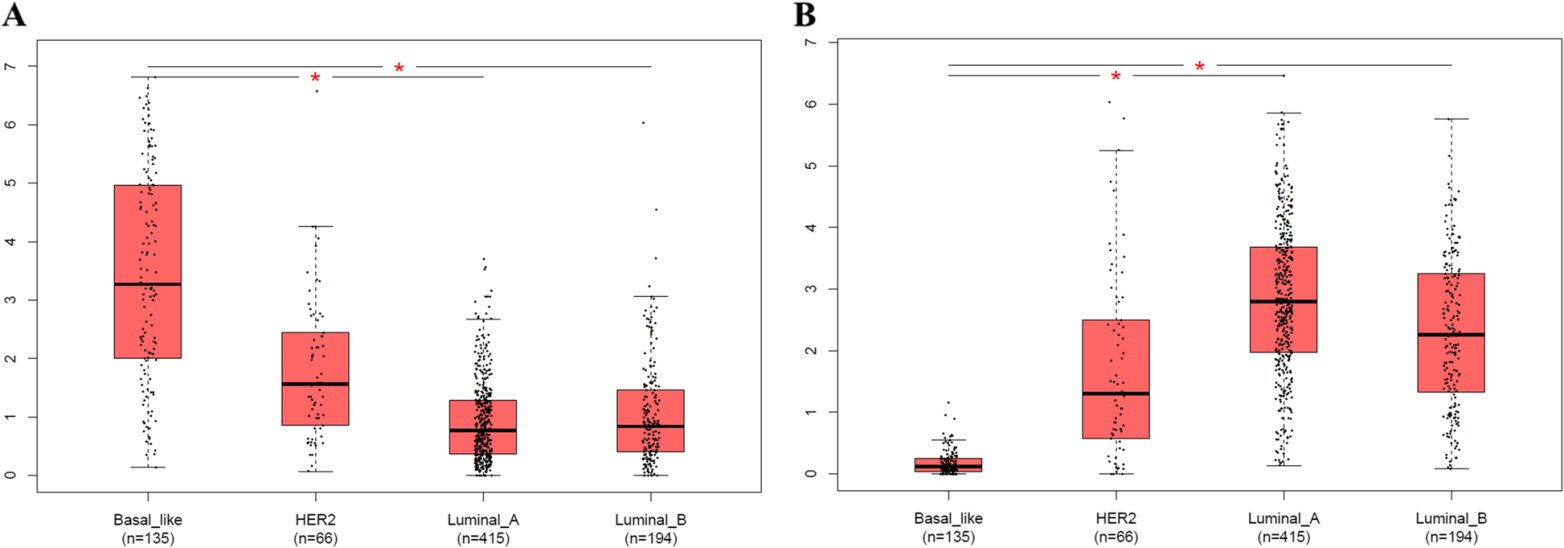
The expression distributions of GAL and TTC36 in breast cancer molecular subtypes. **A** GAL expression. **B** TTC36 expression. *P value < 0.05

These prognostic DEGs were further explored with the specificity of BC molecular subtypes with all TNM stages. The results revealed that the GAL and TTC36 genes were relatively specific to basal-like BC (Fig. 4). Compared to other BC molecular subtypes, GAL was significantly overexpressed in basal-like BC, while TTC36 was significantly downregulated in basal-like BC.

### Expression validation of GAL and TTC36

**Fig. 5:**
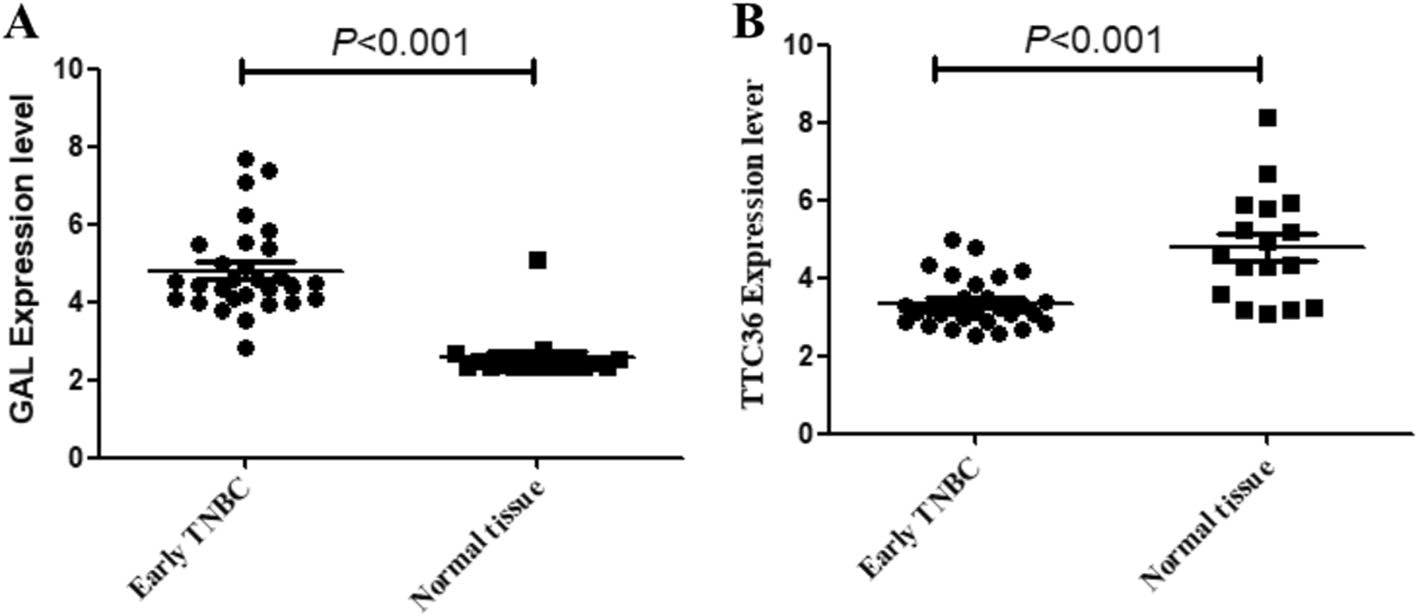
The expression validation of GAL and TTC36 in early-stage basal-like/triple-negative breast cancer. **A** GAL expression. **B** TTC36 expression. TNBC, triple-negative breast cancer

To verify the expression of GAL and TTC36, the gene profiles of 29 cases of early-stage TNBC and 17 normal breast tissues from GEO were analyzed. The results showed that GAL was indeed significantly overexpressed compared to normal breast tissues and that the expression of TTC36 was lower than that in normal breast tissues (Fig. 5).

### Immune infiltration analysis results

**Fig. 6:**
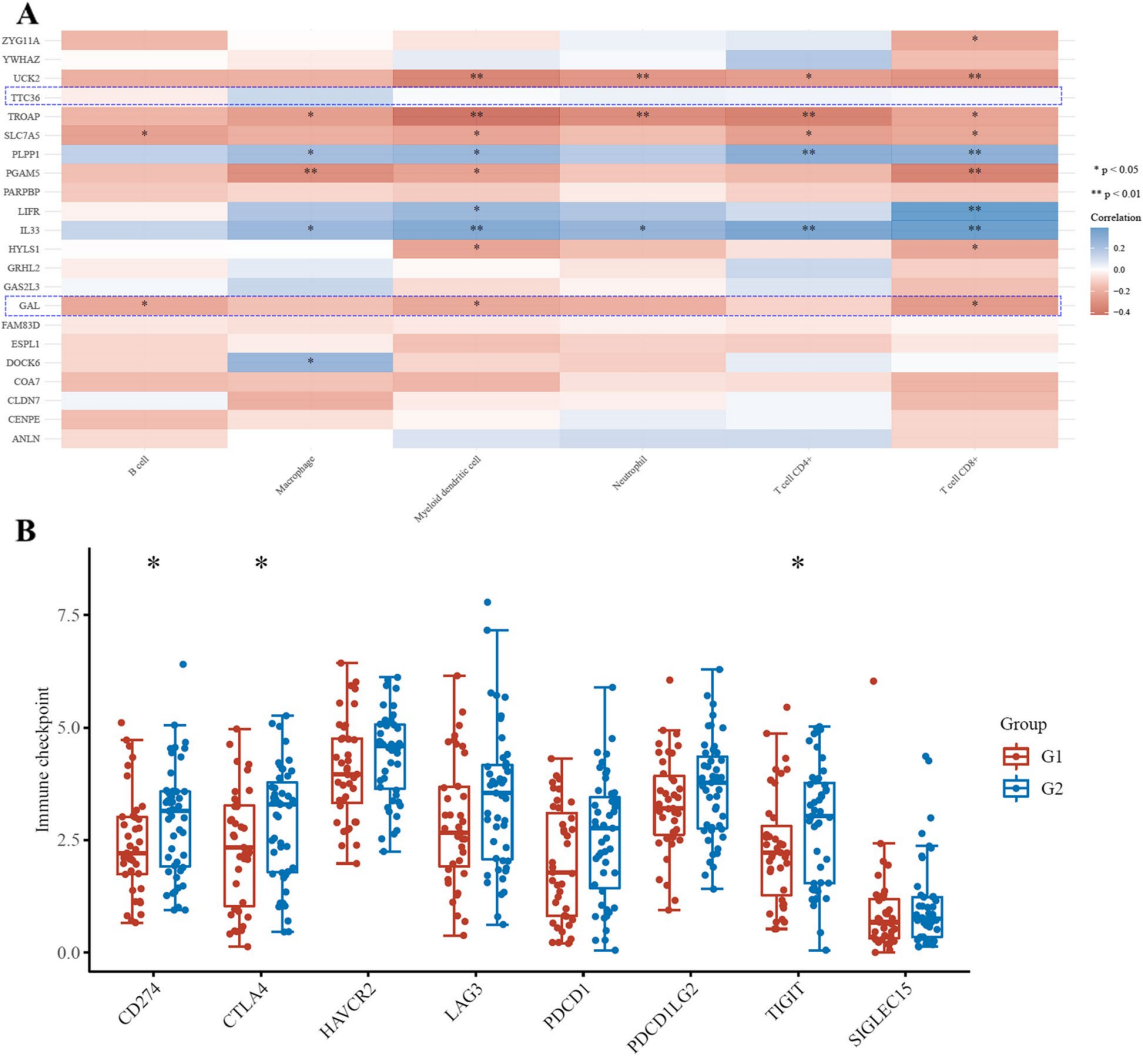
The tumor immune infiltration cell analysis results of differentially expressed genes in early-stage basal-like/triple-negative breast cancer. **A** Immune infiltration cell analysis of all differentially expressed genes. **B** Immune checkpoint analysis of GAL. G1, high expression of GAL; G2, low expression of GAL. *P value < 0.05

The immune infiltration analysis results of prognostic DEGs revealed that in early-stage basal-like BC, the expression of GAL was significantly negatively correlated with the relative ratios of CD8^+^ T cells, B cells, and myeloid dendritic cells (Fig. 6A). However, the expression of TTC36 was not significantly correlated with any immune cells in early-stage basal-like BC. Then, we performed an immune checkpoint analysis of GAL in early-stage basal-like BC. The results showed that the expression distributions of the immune checkpoint genes CD274, CTLA4, and TIGIT were significantly lower in the GAL high-expression group than in the GAL low-expression group (Fig. 6B).

**Fig. 7:**
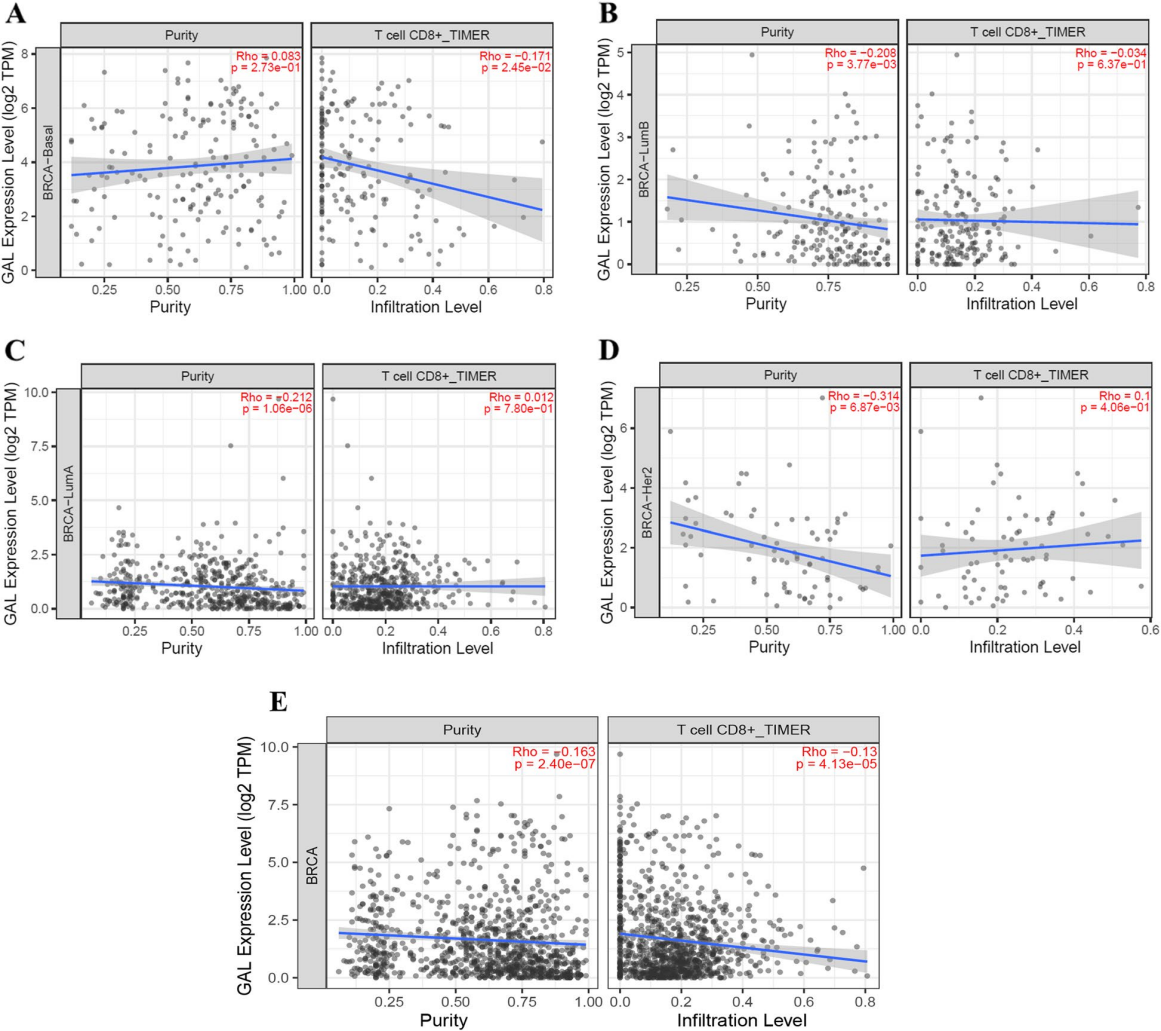
The relationship between GAL expression and the CD8.^+^T cell relative ratio in different molecular subtypes of breast cancer. **A** Basal-like breast cancer. **B** Luminal A breast cancer. **C** Luminal B breast cancer. **D** Her2-overexpressing breast cancer. **E** All types of breast cancer

Furthermore, a BC subtype specificity of GAL immune infiltration analysis was performed in TIMER, and the results showed that only in basal-like BC was the expression of GAL negatively associated with the relative ratio of CD8^+^ T cells (Fig. 7A). There was no significant relationship between GAL expression and the CD8^+^ T-cell relative ratio in luminal A, luminal B, or HER2-overexpressing BCs (Fig. 7B–D). Moreover, there was still a negative correlation between GAL expression and the CD8^+^ T-cell relative ratio in all molecular subtypes of BCs (Fig. 7E).

## Discussion

BC is a commonly prevalent cancer in women and is the second leading cause of female cancer deaths worldwide. Compared to the other molecular subtypes of BC, basal-like/triple-negative BC is the most aggressive type and is characterized by poor outcome and a high rate of early recurrence. Therapy for basal-like/triple-negative BC remains challenging. In this study, we attempted to explore the potential signatures by comparing early-stage basal-like BC and normal breast tissues. We screened a total of 1556 DEGs, with 22 DEGs associated with prognosis. Among these DEGs, GAL and TTC36 were eventually found to be specific to basal-like/triple-negative BC. However, only GAL was proven to be closely related to immune CD8^+^ T cells, which indicated a better response to immune checkpoint therapy.

The GAL gene is highly expressed in enteric nervous tissues and encodes a neuroendocrine peptide, namely, galanin and galanin message-associated peptide (GMAP) [19]. GMAP is part of the innate immune system, modulating natural killer cell function [20, 21]. GAL and its receptors might contribute to the development of gastric cancer [22]. Moreover, the overexpression of GAL was reported close to relapse, micro-metastases, and recurrence in colorectal cancer and eventually resulted in poor prognosis [23]. GAL and its receptor GalR1 might have novel potential for overcoming chemotherapy resistance though mitogen-activated protein kinase signaling and the insulin signaling pathway in colorectal cancer [24]. However, it was also reported that high expression of GAL was associated with suppression of cell proliferation and then promoted tumor cell apoptosis in colorectal cancer [25]. In neuroblastoma, GAL might act as an autocrine/paracrine modulator and counteract neuronal differentiation [26].

In this study, GAL was found to be more highly expressed in early-stage basal-like BC than in normal tissues. Furthermore, overexpression of GAL in basal-like BC was negatively correlated with immune cell infiltration. Although there were significant associations between GAL expression and the CD8^+^ T cell relative ratio in all molecular BC subtypes, the expression of GAL was not significantly related to the CD8^+^ T cell relative ratio in other molecular BC subtypes. The number of tumor CD8 + T cells was reported to be significantly associated with the efficacy of immune checkpoint therapy in various cancers [27]. In TNBC, a high tumor CD8^+^ T-cell score was significantly associated with high expression of multiple immune checkpoint molecules and better survival [28, 29]. Thus, we speculated that the expression of GAL might be associated with the response to immune checkpoint therapy by affecting CD8^+^ T cells in TNBC. We did not find related previous reports about GAL expression in BC. In the last decade, immunotherapy has been extensively investigated and developed to improve the prognosis of TNBC. For instance, it was reported that approximately 20% of TNBC patients expressed PD-L1, which might be involved in cancer immunoediting and improve the response to chemotherapy [30]. Furthermore, immune checkpoint inhibitors have also shown good efficacy in clinical trials [31]. In this study, GAL was found to be a potential and specific biomarker for predicting the response to immune checkpoint therapy in TNBC.

Another gene, TTC36, was also specific to basal-like BC but had no significant correlation with tumor-infiltrating immune cells in this study. TTC36, also known as HBP21, encodes three tetratricopeptide repeats that have a role in interacting with heat shock protein 70. TTC36 was reported to be a tumor suppressor in gastric cancer and hepatocellular carcinoma though the Wnt-β-catenin signaling pathway and promotion of cell apoptosis [32, 33]. Contrary to the results of this study, a previous study revealed that TTC36 was highly expressed in breast cancer tissue [34]. The main reason for this was that this study was focused on exploring early-stage TNBC, not all BC subtypes.

Although various studies had aimed to explore potential or novel biomarkers to predict the prognosis of BC [35, 36], there were still no effective prognostic biomarkers for TNBC. Furthermore, previous related bioinformatic studies on TNBC were performed and revealed several genes of interest that researchers claimed to play an important role in tumorigenesis and are associated with overall survival in TNBC [37–39], this study was mainly focused on early-stage TNBC and was intended to screen basal-like BC-specific genes. However, there are still some limitations in this study. First, the number of early TNBC patients included in this study remained relatively low. Second, the detailed mechanism between GAL expression and tumor immune CD8^+^ T cells is still unclear. Third, the predictive value of GAL in the immunotherapy of early-stage TNBC lacks clinical data. Thus, further research is needed to revalidate the results.

## Supplementary Information

**Additional** **file** **1:** **Figure S1.** The expression distributions of prognostic associated differently expressed genes in breast cancer molecular subtypes. *, *P* value<0.05.

**Additional** **file** **2:** **Figure S2.** The expression distributions of prognostic associated differently expressed genes in breast cancer molecular subtypes. *, *P* value<0.05.

## Data Availability

All data generated or analyzed during this study are included in this published article and its supplementary information files.

## Abbreviations

BC: Breast cancer
TCGA: The Cancer Genome Atlas
PPI: Protein–protein interaction network
TROAP: Trophinin-associated protein
CLDN7: Claudin 7
GAS2L3: Growth arrest specific 2-like 3
GAL: Galanin and GMAP prepropeptide
ZYG11A: Zyg-11 family member A, cell cycle regulator
UCK2: Uridine-cytidine kinase 2
ANLN: Anillin actin binding protein
PLPP1: Phospholipid phosphatase 1
PGAM5: PGAM family member 5, mitochondrial serine/threonine protein phosphatase
GRHL2: Grainyhead-like transcription factor 2
FAM83D: Family with sequence similarity 83 member D
HYLS1: CHYLS1 centriolar and ciliogenesis associated
SLC7A5: Solute carrier family 7 member 5
IL33: Interleukin 33
CENPE: Centromere protein E
YWHAZ: Tyrosine 3-monooxygenase/tryptophan 5-monooxygenase activation protein zeta
COA7: Cytochrome c oxidase assembly factor 7
LIFR: LIF receptor subunit alpha
ESPL1: Extra spindle pole bodies like 1, separase
PARPB: PARP1 binding protein
TTC36: Tetratricopeptide repeat domain 36
DOCK6: Dedicator of cytokinesis 6
DEGs: Differentially expressed genes
GO: Gene ontology
KEGG: Kyoto Encyclopedia of Genes and Genomes
DAVID: Database for Annotation, Visualization and Integrated Discovery
OS: Overall survival
TNBC: Triple-negative breast cancer
