# Supplementary figures and images for: Screening potential immune signatures for early-stage basal-like/triple-negative breast cancer
Source: World J Surg Oncol. 2022 Jun 24;20:214. doi: 10.1186/s12957-022-02683-2 (PMC9229513; doi:10.1186/s12957-022-02683-2)

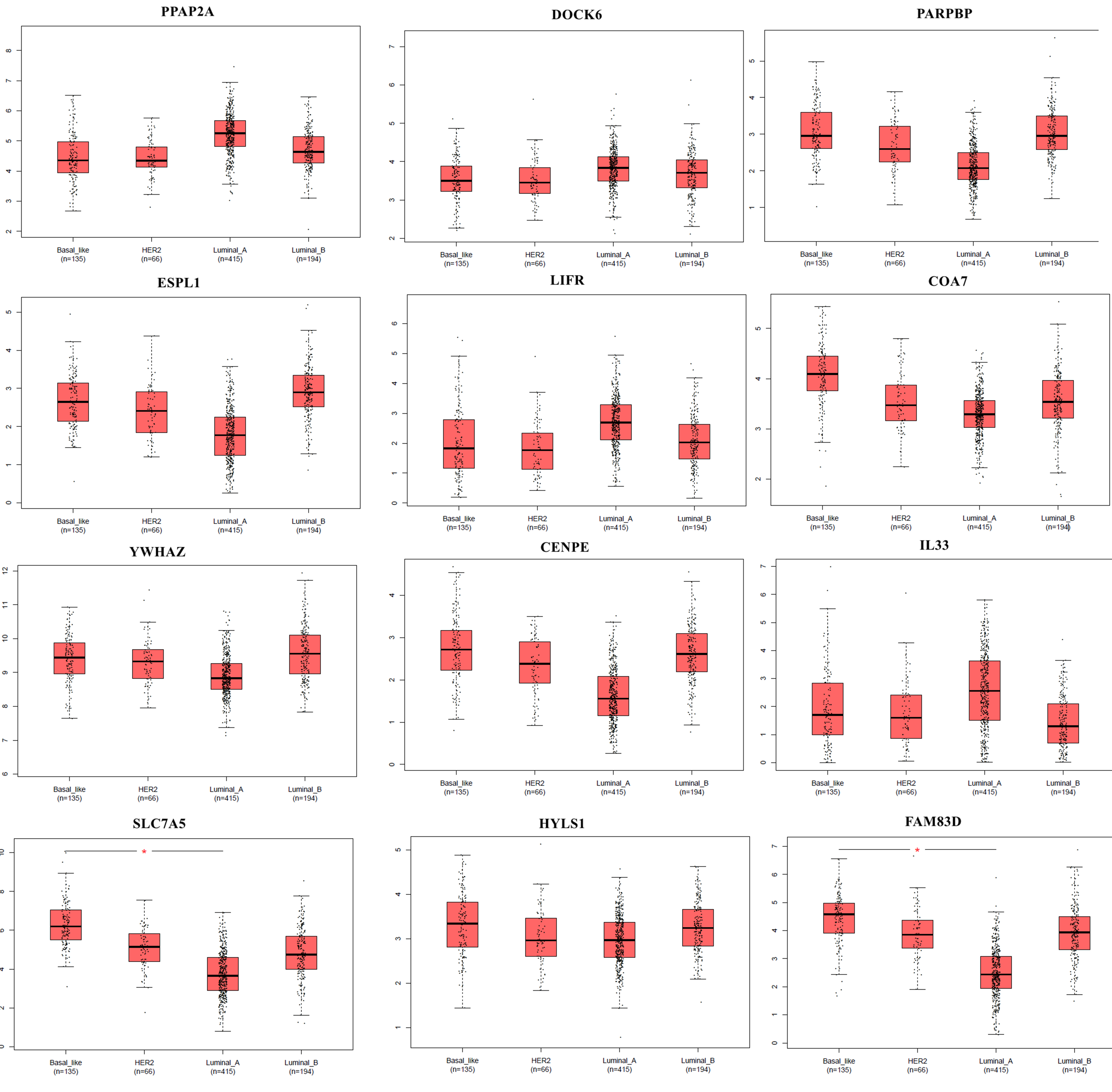

Supplement: Supplementary file 1 — Additional file 1: Figure S1. The expression distributions of prognostic associated differently expressed genes in breast cancer molecular subtypes. *, P value<0.05. [file 12957_2022_2683_MOESM1_ESM.tif]

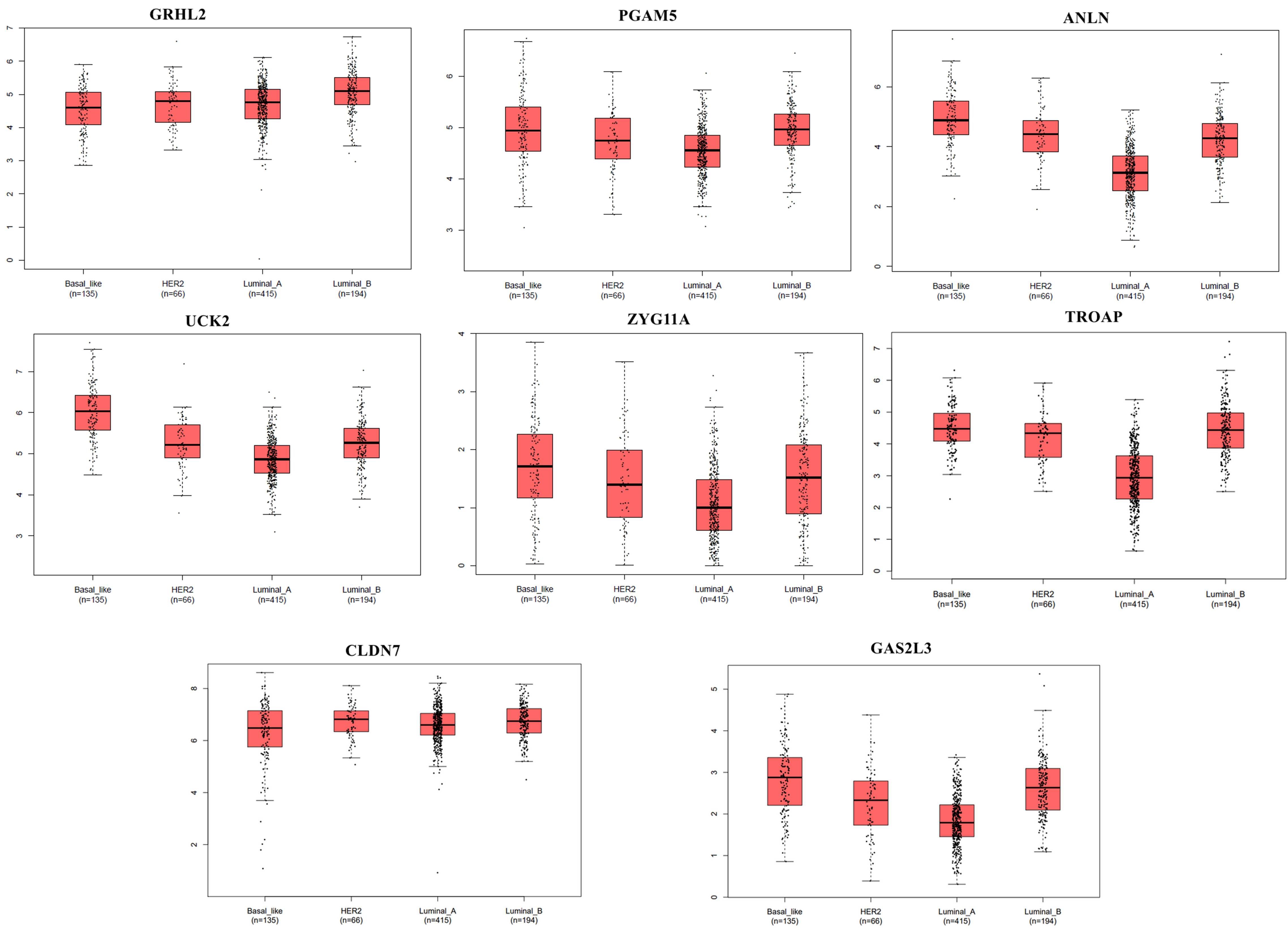

Supplement: Supplementary file 2 — Additional file 2: Figure S2. The expression distributions of prognostic associated differently expressed genes in breast cancer molecular subtypes. *, P value<0.05. [file 12957_2022_2683_MOESM2_ESM.tif]
